# Differentiation Syndrome in Promyelocytic Leukemia: Clinical Presentation, Pathogenesis and Treatment

**DOI:** 10.4084/MJHID.2011.048

**Published:** 2011-10-24

**Authors:** E.M. Rego, G.C. De Santis

**Affiliations:** National Institute of Science and Technology in Stem Cell and Cell Therapy, Division of Oncology/Hematology, Department of Internal Medicine, Medical School of Ribeirão Preto, University of São Paulo

## Abstract

Differentiation syndrome (DS) represents a life-threatening complication in patients with acute promyelocytic leukemia (APL) undergoing induction therapy with all-trans retinoic acid (ATRA) or arsenic trioxide (ATO). It affected about 20–25% of all patients and so far there are no definitive diagnostic criteria. Clinically, DS is characterized by weight gain, fever not attributable to infection, respiratory distress, cardiac involvement, hypotension, and/or acute renal failure. At the histological point of view, there is an extensive interstitial and intra-alveolar pulmonary infiltration by maturing myeloid cells, endothelial cell damage, intra-alveolar edema, inter-alveolar hemorrhage, and fibrinous exsudates. DS pathogenesis is not completely understood, but it is believed that an excessive inflammatory response is the main phenomenon involved, which results in increased production of chemokines and expression of adhesion molecules on APL cells. Due to the high morbidity and mortality associated with DS, its recognition and the prompt initiation of the treatment is of utmost importance. Dexamethasone is considered the mainstay of treatment of DS, and the recommended dose is 10 mg twice daily by intravenous route until resolution of DS. In severe cases (respiratory or acute renal failure) it is recommended the discontinuation of ATRA or ATO until recovery.

## Introduction

Contemporary treatment of acute promyelocytic leukemia (APL) consists of a combination of all-*trans* retinoic acid (ATRA) with anthracycline-containing chemotherapy, or arsenic trioxide (ATO). These modalities of treatment lead to complete remission rates greater than 90% and cure rates of approximately 80%, in contrast with results reported before introduction of ATRA, in which disease free survival after 3 years were inferior to 20%.^1^ The introduction of ATRA, which belongs to a class of chemical compounds related to vitamin A known as retinoids, to treat APL in the 1980s revolutionized the concept of treatment of cancer, of which, besides the aim to destroy by chemotherapy the pathologic cells, there is the objective to enforce its maturation.^2^ This latter process culminates with cell death. ATRA, when administered in pharmacological doses, triggers APL cells to differentiate into mature granulocytes. The molecular process is not fully known but the main aspect involves the degradation of the complex PML-RARα, a phenomenon that unleashes the mechanisms required for terminal maturation.

Besides ATRA, arsenic trioxide (ATO) is an important agent that is being used for APL treatment since the early 1990s. ATO, or white arsenic, is one of the three forms in which arsenic exists.^3^ ATO is one of the oldest drugs known to man.^4^ Despite its reputation of being a poison and carcinogenic agent, ATO is usually well tolerated. This drug degrades PML-RARα by targeting its PML moiety (it also degrades normal PML). ATO provokes apoptosis when used at high concentration (1–2 × 10^−6^ M), or partial maturation of APL cells when used at low concentration (0.25–0.5 × 10^−6^ M) and for a longer period of time. Through these actions it improves the clinical outcome of refractory, relapsed or newly diagnosed APL.^5^,^6^ Both ATRA and ATO, alone or in combination, can trigger differentiation syndrome (DS), a relatively common complication of APL treatment previously named retinoic acid syndrome.^7^

Clinically, DS is characterized by weight gain, fever not attributable to infection, respiratory distress, cardiac involvement, hypotension, and acute renal failure.^7^ DS is potentially fatal and its recognition and treatment is of utmost importance. Histological analysis of patients who died of DS revealed extensive interstitial and intra-alveolar pulmonary infiltration by maturing myeloid cells, endothelial cell damage intra-alveolar edema, inter-alveolar hemorrhage, and fibrinous exsudates.^7^,^8^

Regarding DS incidence, most studies reported that approximately one fourth of APL patients receiving ATRA as induction therapy,^9^ but depending on the criteria employed; this incidence was lower. For instance, Mandelli et al, from GIMEMA, considered DS when at least five symptoms were present, and as a result DS incidence was of only 2.5% (6/240).^10^

As mentioned previously, ATO is also associated with DS with a similar frequency.^11^ However, the combination of ATRA and ATO was reported to have induced DS in only 16% (13/82) of patients.^12^ This finding is not a surprise, as it was demonstrated that the combination ATRA/ATO was associated with fewer complications than ATRA or ATO alone.^13^

## Clinical Picture

Table 1 lists the main symptoms of DS and their respective frequency. De Botton et al reported in a large series of patients who developed DS revealed that respiratory signs/symptoms and fever were the most frequent clinical presentation of DS. These authors described 413 patients with newly diagnosed APL, 64 (15%) of which developed DS (median at day 7), and 9 (14%) patients died of it.^14^ Respiratory involvement was reviewed recently.^15^ Its most common manifestations are pulmonary infiltrate, pleural effusion and respiratory distress. Cardiac involvement is more commonly characterized by pericardial effusion, but it can also present as chest pain typical of coronary obstruction.^16^ Rarer clinical presentations such as musculoskeletal symptoms were also reported.^17^ There is no pathognomonic clinical sign or laboratory test to diagnose DS. For this reason, sometimes DS can be misdiagnosed or confounded with other concurrent medical condition, such as infection and heart failure. It was suggested to consider DS when at least three of the following signs or symptoms are present: fever, weight gain, respiratory distress, pulmonary infiltrates, pleura or pericardial effusions, hypotension, and renal failure.^9^

Among the conditions that clinically resemble DS are: acute respiratory distress syndrome (ARDS),^18^ hyperkeukocytosis/leukostasis in AML^19^ and engraftment syndrome after hematopoietc stem cell transplantation.^20^ In all these conditions, there is an organ infiltration, especially the lungs, by granulocytes or leukemic cells activated by inflammatory cytokines, growth factors or differentiation agents. Curiously, and certainly not fortuitously, some of them can be successfully treated with corticosteroids.

## Pathogenesis

The molecular mechanisms leading to DS development are not fully known, but it is believed that an excessive inflammatory response is the main phenomenon involved. This inflammatory response is provoked by leukemic cells in the process of differentiation, which results in increased production of chemokines and expression of adhesion molecules on APL cells. The inflammation would result in organ infiltration by blast cells, especially the lungs, capillary-leak syndrome and organ failure. The mechanism must be similar to what occur with normal granulocytes recruited to sites with inflammation, in which the circulating leukocytes are captured by the endothelial cells (EC) on which occurs the process called rolling (mediated by selectins), followed by their firm attachment to EC (mediated by integrins), and finally, their transendothelial migration into the tissue.^21^ Leukocyte transmigration requires secretion of proteases (metalloproteases and neutrophil elastase) that disrupts the endothelial barrier. As a consequence there is extravasation of fluids into alveolar space.^22^,^23^

A large array of chemokines has their production greatly increased by treatment of APL cells with ATRA.^24^,^25^ The migration of blast cells to lungs is triggered by increased specific chemokines production by alveolar epithelial cells. APL cells are the attracted to lungs where they transmigrate into tissue and alveolar space. APL cells also contribute to chemokine production, which increase further blast recruitment. Administration of corticosteroids suppresses chemokine production by alveolar cells and then abrogates lung infiltration. Besides corticosteroids, neutralizing antibodies was able to reduce in vitro transmigration of leukemic cells (NB4) towards alveolar epithelial cells (A549).^26^,^27^

In addition to chemokine increased production, it was demonstrated by our group and others that treatment with ATRA up-regulated the expression of adhesion molecules on blast cells and on endothelial cells, and that dexamethasone counteracted the effects of ATRA.^28^–^30^ Our group has demonstrated that NB4 cells (an APL cell lineage) and primary APL cells treated with ATRA enhanced their expression of CD54 and CD18. Moreover, mice injected with APL cells previously exposed to ATRA had the lungs infiltrated by blasts, demonstrated by increased myeloperoxidase (MPO) activity. The infiltration however could be blocked by dexamethasone and neutralizing antibodies against adhesion molecules CD54 and CD18. Furthermore, knockout mice for CD54 had not increased MPO activity in lungs after injection of treated APL cells. These findings demonstrated the main role certain adhesion molecules have in DS development and unveil part of the mechanisms of action of corticosteroids in treatment of this syndrome.^30^

## Risk Factors

Risk factors for developing DS are controversial. Vahdat et al have shown that peripheral blood leukocyte count peak at the onset of DS symptoms.^31^ In agreement of this finding, Tallman and colleagues reported that in 44 of 167 (26%) patients with APL receiving ATRA, the median white blood cell (WBC) count at diagnosis was 1,450/μL, and 31,000/μL at the time the syndrome was diagnosed. However, neither the initial leukocyte count nor the rate of rise in leukocyte counts on days preceding DS correlated with its incidence. The European trial showed that patients with WBC > 5 × 10^9^/L at presentation and DS tended to require mechanical ventilation more frequently than patients with lower WBC.^14^

Regarding the expression of myeloid associated markers, in the study of Vahdat et al. the basal expression of CD13 (amino-peptidase N) was highly associated with both development of DS as well as with elevated leukocyte count. However, others and our group did not find correlation between basal WBC count or immune-phenotype characteristics (expression of CD33, CD13 and CD117) with incidence of DS ().^32^

Recently, Montesinos et al reported the outcome of 739 APL patients treated with ATRA and idarubicin and found that variables predictive of severe DS included WBC > 5 × 10^9^/L, abnormal serum creatinine levels, FTL3-ITD mutation, the microgranular subtype, the short *PML-RARA* isoform, and male sex.^33^ However, in a multivariate analysis, only WBC counts and serum creatinine levels were significant. Moreover, with respect to APL morphological subtype, the microgranular variant (M3V) was found by others to protect against DS by the US Intergroup study.^8^ However, recently, Tallman and colleagues, in a large joint study of the North American Intergroup and the PETHEMA Group, found that the incidence of DS was 26% in M3V and 25% for the classical APL (P= 0.66).^34^

Another factor that seems to influence the incidence of DS is the timing of initiation of chemotherapy. De Botton et al have shown that early onset of chemotherapy can reduce the incidence of DS in newly diagnosed APL with low WBC count at presentation (< 5 × 10^9^/L). The incidence of DS in the ATRA with concurrent chemotherapy arm was lower than in the arm in which chemotherapy was not started concurrently (9.2% and 18% respectively, *P*= 0.035).^35^

Dore et al reported an association between development of DS and the AA genotype at Codon 469 of *ICAM-1*, which suggests that susceptibility to DS in APL patients may be influenced by genetic variation in adhesion molecule loci.^36^ Finally, it was recently demonstrated that high body mass index is an independent predictor of DS.^37^

## Management and Outcome

As DS can have a subtle clinical picture at presentation but progress rapidly, it is of utmost importance to be aware of this complication and initiate therapy as soon as it was suspected. Initial measures involves ventilatory and blood pressure support. Dexamethasone is considered the mainstay of treatment of DS, and should be administered at the first sign or symptom of this syndrome. The dose recommended is 10 mg twice daily by intravenous route until resolution of DS, after which the dose can be progressively reduced in the next few days or weeks. There is no need to discontinue ATRA if DS is not severe. However, in severe cases (respiratory or acute renal failure) it seems reasonable to discontinue the drug until clinical recovery, when ATRA could then be restarted (Table 2). In the study by Tallman et al. ATRA was discontinued in 36 of the 44 patients (82%) that developed DS and resumed in 19 of the 36 patients (53%). However, DS recurred in 3 (16%) of those 19 patients.^8^

Prophylactic administration of steroids is controversial. There is no evidence of its benefit in reducing DS incidence or severity (Sanz et al, 2009). For this reason, this approach cannot be recommended at moment, despite the report that the prophylactic use of corticosteroids from the start of ATRA reduced the incidence of severe DS, but not its mortality.^33^

Some patients have DS that are refractory to corticosteroids. There are yet no widely accepted alternative to it. It seems reasonable to employ in future agents that block migration, adhesion or transmigration of APL cells. A few years ago, Kawasaki and colleagues administered sivelestat, a small molecule that inhibits neutrophil elastase, and that has been shown to be effective in animal models of ARDS/ALI, reported its successful use in two patients with DS.^38^

The frequency of death due to DS varied from 7.8% to 33% in clinical trials.^7^,^39^ In the study by De La Serna et al DS was responsible for approximately one fifth of induction deaths, which occurred at a median of 17^1^–^26^ days of starting induction.^40^ Taken together these results suggest that there was a decrease in DS mortality in recent years, probably due to a more prompt recognition of its symptoms and signals and early introduction of therapeutic measures.

## Conclusions

DS is an unpredictable complication of treatment of APL with ATRA or ATO, and occurs usually after a few days or weeks of initiation of induction therapy. It is extremely uncommon during maintenance treatment. There are no laboratory tests or clinical exams specific for DS. Considered alone, demonstration of diffuse opacity of lungs on Rx suggestive of edema is perhaps the most suggestive exam for DS.

Treatment consists of ventilation support and administration of steroids by intravenous route for a few days or weeks after clinical resolution. In severe cases (respiratory or acute renal failure) it seems reasonable to discontinue ATRA until clinical recovery, when it could then be restarted. Concurrent chemotherapy can be useful to reduce incidence and severity of DS, despite its potential dangerous effect on blood hemostasis. Despite the fact that, in the last years, concurrent chemotherapy has reduced the incidence and severity of DS, the most important action to reduce DS morbidity and mortality remains the early recognition of its symptoms, and institution of supportive measures and treatment with dexamethasone.

## Figures and Tables

**Table 1:** Clinical picture of DS: approximate frequency of signs and symptoms.

Respiratory distress/pulmonary infiltration	80–90%
Fever	80%
Weight gain (> 5 Kg)	50%
Pleural effusion	50%
Renal failure	40%
Pericardial effusion	20%
Cardiac failure	15–20%
Hypotension	10–15%

**Table 2:** Measures at suspicion of DS

Chest x-ray, renal function (creatinine and urea), hepatic function (amino transferases and bilirubin), blood cell counts, coagulation tests, oxygen saturation
Weight monitoring
Ventilatory support/O_2_ supplementation
Blood pressure maintenance measures
Fluid restriction (renal failure)
Steroid administration at firs suspicion: dexamethasone 10 mg twice daily until clinical resolution, then tapered dose for a few days
Suspend ATRA or ATO in severe cases, which can be restarted after clinical improvement. If DS recurs after restart, ATRA must be definitively discontinued during induction.
